# Inlay total shoulder arthroplasty in the weight-bearing shoulder of wheelchair-bound patients with paraplegia. A case report

**DOI:** 10.1016/j.ijscr.2024.110373

**Published:** 2024-10-01

**Authors:** Zachary A. Blashinsky, Steven Latta, Matthias R. Schurhoff, Luis A. Vargas, John E. Zvijac, John W. Uribe

**Affiliations:** a Baptist Health Orthopedic Care, Baptist Health South Florida, Coral Gables, FL, USA; b Florida International University Herbert Wertheim, College of Medicine, Miami, FL, USA

**Keywords:** Glenohumeral joint, Shoulder, Arthroplasty, Paraplegic, Osteoarthritis, Orthopedics, Sports medicine, Case report

## Abstract

**Introduction and importance:**

Paraplegic patients' activities of daily living (ADLs) involve the use of manual wheelchairs that increase the stress on the shoulder joint. Patients with advanced glenohumeral changes are often resistant to conservative measures and may require surgical interventions. The longevity of a Total Shoulder Arthroplasty is largely unknown in paraplegics due to a lack of studies investigating outcomes in these patients.

**Case presentation:**

We examined the outcome of two paraplegic patients following inlay total shoulder arthroplasty (iTSA) with a non-spherical humeral head and glenoid inlay replacement.

**Clinical discussion:**

Two patients with paraplegia and advanced degenerative joint disease who underwent iTSA were seen at regular follow-up intervals to assess range of motion, strength, and patient-reported outcome measures. Radiographic imaging was utilized to monitor slipping, lateralization, and degradation of the joint space.

**Conclusion:**

Both patients have shown significant increases in strength, ROM, and PROMs. iTSA proved efficacious in paraplegic patients utilizing a manual wheelchair.

## Highlights

• Patients with paraplegia are at increased risk of shoulder pathology as the shoulder is a weight-bearing joint of the body.
• Patients with advanced degenerative glenohumeral arthritis lack clear treatment recommendations.
• Two patients with paraplegia had non-spherical humeral head total shoulder arthroplasty with an inlay glenoid component.
• Both patients showed improved PROs, ROM, and no signs of degradation on radiographic assessments.
• iTSA proved effective in treating patients with paraplegia in short and long-term follow-up.

## 1 Introduction

Patients with paraplegia have unique activities of daily living (ADLs) that frequently involve the use of manual wheelchairs that increase the stress on the shoulder joint [1]. Given that approximately 3.7 million individuals in the United States (U.S.) rely on wheelchairs [2], it is important to prioritize care that supports their ADLs. Shoulder pain and pathology are more prevalent in patients with paraplegia as the shoulder becomes a major weight-bearing joint of the body [3,4]. Shoulder pain is reported to be as high as 70 % in manual wheelchair users [5]. In previous studies, successful reduction of shoulder pain has been demonstrated with exercise intervention; however, patients with advanced degenerative glenohumeral changes are often resistant to conservative measures and may require surgical interventions.

Treating shoulder injuries in wheelchair-bound patients requires careful consideration to optimize recovery time and preserve independence. The longevity of a total shoulder arthroplasty (TSA) is largely unknown in patients with paraplegia due to the additional stress on the joint and the lack of studies investigating the outcome in these patients [6]. In this case report, we examine the outcome of two patients with paraplegia following inlay total shoulder arthroplasty (iTSA) with a non-spherical humeral head and glenoid inlay replacement. The information presented in this report is in line with the SCARE checklist [7].

## 2 Case report

The first patient (patient I) is a 68-year-old male with paraplegia who sustained a traumatic C5 and C6 fracture 40 years prior to total shoulder replacement. Before the accident, he was right-hand dominant but has learned to become ambidextrous. The patient presented to clinic with bilateral shoulder pain that had progressively worsened for two years and reported a significant loss of function in both shoulders.

**Fig. 1:**
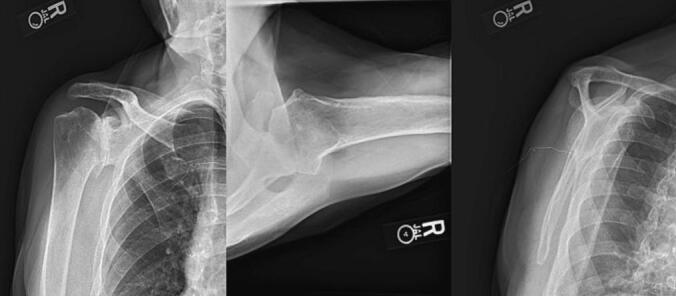
Pre-operative AP, axillary, and scapular radiographs of right shoulder demonstrating severe glenohumeral osteoarthritis in patient I.

On physical examination, passive range of motion (ROM) was 170° of forward elevation and 60° of external rotation with a 5/5 rotator cuff strength on the left. Passive range of motion on the right was limited to 150° of forward elevation and 30° of external rotation. The patient maintained 5/5 supraspinatus and external rotator strength. However, he reported a loss of function with normal ADLs. Radiographs revealed advanced degenerative joint disease in both shoulders, with more severe degeneration of the right shoulder as seen in Fig. 1. Radiographic assessment using Walch classification showed a right A2 shoulder, a Samilson-Prieto Classification stage III, and subchondral glenoid cysts. Treatment options were discussed with the patient, who elected to undergo arthroscopic debridement of the left shoulder followed by staged iTSA on his right shoulder.

**Fig. 2:**
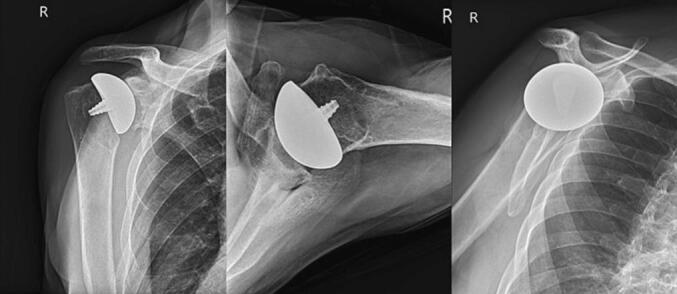
One-year postoperative AP, axillary, and scapular radiographs of the right shoulder in patient I.

The patient returned for iTSA on the right side three months following left shoulder arthroscopy. The procedure was performed in a beach chair position using a deltopectoral approach with a subscapularis tenotomy. The glenohumeral joint was exposed and both joint surfaces showed complete loss of articular cartilage and multiple eburnations. A large osteophyte was removed from the humerus's inferior surface, and a cyst was excised medial to the bicipital groove. Following standard technique [8], the humeral head and glenoid were prepared for and a 58 × 54 mm non-spherical humeral head and 20 × 25 mm inlay glenoid components were used. The humeral head was reduced, full range of motion was confirmed, the subscapularis was repaired, and the incision was closed with staples. The patient required no transfusions, and no complications were experienced in the intraoperative or immediate postoperative period. One-year postoperative radiographs confirmed appropriate implant placement (Fig. 2). The patient was discharged with at-home exercise instructions on the same day of the operation.

Patient had regular scheduled follow-up at five weeks, three months, and six months time points, ROM and strength continued to improve (Table 1). The patient reported several recent falls at the one-year time point but denied any injury to the shoulder. At two-year follow-up, the patient demonstrated flexion of 165°, extension of 50°, abduction of 120°, external rotation of 90°, and internal rotation of 90°. Post-operative radiographic assessment performed at 30 months showed periprosthetic radiolucency of less than 1 mm in the anterior-posterior plane of the humeral head. No component failure or joint loosening was visualized.

**Table 1:** Patient I range of motion and muscle strength timeline.

Time	Forward elevation (flexion)	External rotation	Strength
Preoperative	150°	30°	
Intra-operative	150°	70°	
5-week	150°	60°	4/5 Supraspinatus and external rotation
3-month	130°	30°	5/5 Supraspinatus and internal rotation 5/5 external rotation
6-month	165°	60°	5/5 All rotator cuff muscles
1-year	165°	60°	5/5 All rotator cuff muscles
2-year	165°	90°	5/5 All rotator cuff muscles

**Fig. 3:**
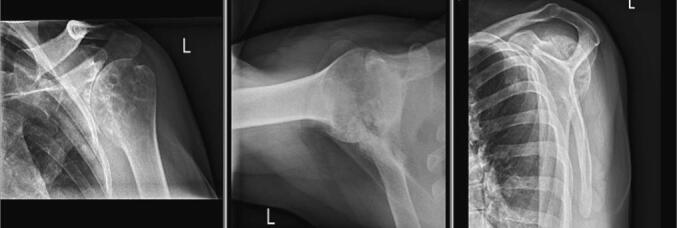
Preoperative radiographic images of left shoulder showing degenerative joint disease in patient II.

Patient II is a 64-year-old male with T3 complete spinal cord injury following an accident in the Vietnam War. He presented with a one-month history of pain in his left, non-dominant shoulder. Previous corticosteroid injections did not provide any symptom relief. He reported the pain was constant and exacerbated by lifting and movement. The patient complained of decreased mobility, difficulty sleeping, and paresthesia in the arms. The drop arm test was positive, and abnormal strength tests in the supraspinatus, and external rotation were observed. Left arm forward elevation was 60°, and external rotation was zero degrees. The diagnosis of glenohumeral osteoarthritis with a torn rotator cuff was made based on physical examination and preoperative radiographic imaging (Fig. 3). Left arm radiographic assessment showed Walch A2 glenoid morphology, Samilson-Prieto Stage III glenohumeral osteoarthritic changes, and subchondral cysts in both the humerus and the glenoid. Following a discussion of treatment alternatives, the patient agreed to undergo left iTSA with repair of the supraspinatus tendon.

**Fig. 4:**
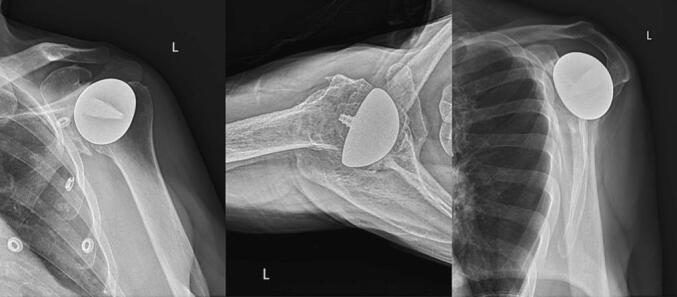
10-year postoperative radiographic imaging of iTSA in patient II.

The humeral head and glenoid were devoid of articular cartilage. Significant eburnation with osteophyte formation inferiorly in the humeral neck and a tear of the supraspinatus insertion on the greater tuberosity were found. Periarticular osteophytes were removed, a non-spherical humeral head, 58 × 54 mm was utilized, and a 20 × 25 mm inlay glenoid was placed into the inferior glenoid vault. The supraspinatus tear was repaired with minimal tension using the single-row technique. Three-month postoperative follow-up showed flexion of 110°, external rotation of 30°, and strength assessment showed 5/5 strength of the deltoid, and 4/5 strength on external and internal rotation, and supraspinatus. Post-operative functional assessments were measured at each follow-up clinic visit and documented in Table 2. Radiographic assessments at 10-year-follow-up showed no periprosthetic radiolucency, subsidence, or dislocation (Fig. 4). A subchondral cyst of the glenoid was noted.

**Table 2:** Patient II range of motion and muscle strength timeline.

Time	Forward elevation (flexion)	External rotation	Strength
Preoperative	60 (active)	0	Positive drop arm test 4/5 Strength in supraspinatus, and external rotation
4-week	80		
3-month	110	30	5/5 Deltoid; 4/5 external rotation, internal rotation, supraspinatus
6-month	120	30	
1-year	130	30	Strength 5/5 on all tests
2-year	130	30	Strength 5/5 on all tests
3-year	130	30	Strength 5/5 on all tests
10-year	130	40	Strength 5/5 and negative on all tests

## 3 Results

The Patient-reported outcomes (PROs) used to assess both patients included the American Shoulder and Elbow Surgeons Shoulder Score (ASES), the Single Assessment Numeric Evaluation (SANE), and VAS pain (0–10 Worst) and can be seen in Table 3 [9,10]. Western Ontario Osteoarthritis of the Shoulder (WOOS) index was incorporated at the most recent follow-up for patient I [11]. PROs were reassessed at every visit. The PROs for the right shoulder in patient I one month before surgery included ASES of 43.33, SANE of 39.11, and VAS pain of 5/10. Two-year postoperative PROs showed ASES of 90, Shoulder Pain VAS of 0, SANE score of 80, and WOOS total score and percentage of 350 and 82 % respectively.

**Table 3:** Summary of patient-reported outcomes for the right shoulder of patient I.

Outcomes	Preoperative	1-year	2-Year
ASES	43.33	93.33	90
Shoulder Pain VAS	5	0	0
SANE	39.11	80	80
WOOS	n/a	n/a	350 (82 %)

**Fig. 5:**
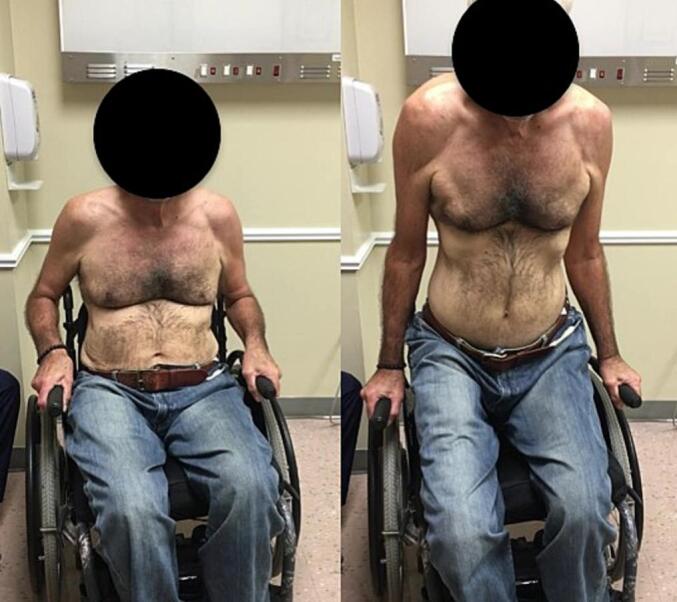
Patient II demonstrates common lift-off movement at 10-year post-operative follow-up.

The PROs for the left shoulder in Patient II one month before surgery included an ASES of 25 and VAS pain of five. Ten-year post-operative PROs showed SANE 90, VAS-pain 0, and ASES 90 (Table 4). The patient was able to demonstrate a lift-off movement commonly performed by wheelchair patients by raising his buttocks off the wheelchair using his arms as shown in Fig. 5. Both patients demonstrated improved and consistent PROs at each post-operative visit.

**Table 4:** Summary of patient-reported outcomes for the left shoulder of patient II.

Outcomes	Preoperative	1-year	2-year	4-year	10-year
ASES	25	93.3	98.3	100	90
Shoulder Pain VAS	5	0	0	0	0
SANE	n/a	n/a	n/a	n/a	90

## 4 Discussion

Wheelchair-dependent patients heavily rely on their upper extremities, especially their shoulders, for mobility and performing ADLs. Functionality is of the utmost importance for this patient population as it is directly correlated with ADLs and transportation. The shoulder joint is subjected to constant stress and functionally becomes a significant weight-bearing joint of the body [6]. The act of lifting one out of the wheelchair by pressing on the arms is a common technique used by people with paraplegia to relieve lower back and buttock pain. This maneuver is associated with more significant shoulder loading and narrowing of the acromial-humeral distance, progressing to a higher incidence of shoulder pain [3]. Early onset shoulder pain and dysfunction is a frequent problem in these patients, causing a significant negative impact on their independence and quality of life. The critical issue associated with surgery in this population is ensuring longevity in the joint while simultaneously attempting to limit postoperative recovery time. Extended recovery in wheelchair-dependent patients may impact their independence when performing ADLs and reduce their quality of life.

Hattrup et al. reported on six patients with paraplegia who underwent shoulder arthroplasty [12]. The study included one humeral head replacement and five TSAs with an average follow-up of 84 months (Range 24–200), resulting in a Neer classification of 1 excellent, 4 satisfactory, and 1 unsatisfactory [12]. Rotator cuff tearing or thinning was visualized in all patients before surgery. Recovery included immobilization for six weeks, and utilization of an aide authorized at weeks eight through ten. Complications were frequent and included pressure ulcers, brachial plexopathy, and humeral head fractures. Additionally, the length of time of immobilization may be debilitating to a wheelchair-bound patient and significantly impact their ADLs.

Another study used the ASES function score to assess pain and function in five female patients with paraplegia undergoing TSA. The score ranges from 0 to 100, with 100 indicating less pain and higher function. The preoperative ASES score in this study was 28 and improved to a mean of 37 after the patients underwent arthroplasty [13]. Out of the five participants, only one patient reported complete pain relief.

A case report on a 69-year-old patient with paraplegia showed that continued manual wheelchair use led to three failed shoulder replacements [14]. The patient underwent TSA, hemiarthroplasty, and a reverse TSA that failed following manual wheelchair use and infection. The patient continued to use a manual wheelchair despite the physician's recommendation and owning an electric wheelchair stating that using anything other than a manual wheelchair made him feel disabled. Finally, the RTSA was replaced with an injection-modeled antibiotic-containing spacer. Patient non-compliance in this case emphasizes the role that independence concerns and psychosocial support have in this unique population.

A retrospective review of the surgical outcomes of rotator cuff tears in wheelchair-bound patients identified five male patients and eight shoulders [15]. All five patients would recommend rotator cuff repair to other spinal cord injury patients. Seven of eight shoulders had improved function to their preinjury level. This case also emphasized the importance of proper patient selection due to the demanding postoperative rehabilitation.

A study of 22 wheelchair-dependent patients who underwent reverse total shoulder arthroplasty (rTSA) found significant improvement in pain but non-significant changes in the range of motion [16]. Although all reported a willingness to undergo surgery again, only three reported excellent outcomes, with 12 reporting satisfactory and another four reporting unsatisfactory results.

Total shoulder arthroplasty with stemless, non-spherical head and inlay glenoid component has shown significant success in patients with advanced shoulder arthritis [8]. Traditional TSA has been correlated with glenoid loosening, leading to abnormal glenoid version angle known as the “rocking horse” phenomenon [17,18]. The non-spherical humeral head component provides a more anatomically correct implant with less volume in the AP plane [19]. The stemless implant prevents stress riser fractures and maintains the option to advance to stemmed arthroplasty [20]. Inlay glenoid reduces lateralization of the joint space compared to onlay glenoid components [21]. A study comparing onlay versus inlay glenoid implants in cadaveric shoulders tested force distribution and loosening with fatigue testing in 16 shoulders [22]. The onlay implant showed greater force distribution and gross loosening on all implants. All inlay glenoid implants were intact without loosening.

Studies have also shown that patients can maintain an active lifestyle and stay involved in high-demand activities, including weightlifting, following this procedure [23]. The shoulder joint in weightlifters endures similar stress to the shoulders in wheelchair-dependent patients and, therefore, could show promising results in the latter population. Yalcin et al. showed a 100 % return to sport in elite weightlifters with 44 % reporting lifting heavier loads compared to preoperative baseline. Cvetanovich et al. recorded PROs and return to activity for 27 shoulders in an active population [24]. Patients experienced improved PROs, reported a rate of 93 % return to work, and a 75 % return to sport.

While many variations of shoulder arthroplasty are available, no comprehensive recommendations have been made for higher-impact sports, including weightlifting, in conventional TSA designs [25]. The lack of literature produces challenges for both patients and surgeons. For example, patients are frequently concerned about losing independence in the immediate postoperative period. At the same time, surgeons may be concerned over implant longevity due to the lack of literature on long-term TSA outcomes in populations where the shoulder functions as a major weight-bearing joint. While surgical complications are common in these patients [14,26], few studies have investigated the long-term outcomes of iTSA in patients with paraplegia. While our study lacks statistical power due to population size, the comparative clinical outcomes for patients compared to the general population are paramount for surgical judgment and the decision to undergo operative management. Recently, a systematic review of clinical outcomes and complications compared upper extremity ambulators (UEAs) to bipedal ambulator control groups [27]. Of 248 UEA patients, 37 (15 %) underwent anatomic TSA, and the majority (79 %) underwent reverse TSA. Assistive devices varied with 38 % reporting wheelchair use. Patients demonstrated significant improvements in PROMs and when compared to control groups, had no significant differences in outcomes. The complication rate was 17 % for UEAs compared to 9.1 % for controls, and the reoperation rate for revision surgery was 7.7 % for UEAs and 4.9 % for controls.

## 5 Conclusion

Inlay total shoulder arthroplasty proved efficacious in two patients with paraplegia utilizing a manual wheelchair in both mid and long-term follow-up. The patients were able to return to ADLs and reported a significant increase in ROM, strength, and stability. Radiographic imaging showed no periprosthetic radiolucency, lateralization, degradation, or component failure. While other techniques for reconstruction have yielded varying results, we believe this technique could be considered a reliable treatment option for patients with paraplegia with similar pathology. Surgical management in patients with paraplegia will allow for greater autonomy and performance of ADLs. Further investigation is needed in this unique population to support patient autonomy and mitigate restrictions of ADLs in the immediate post-operative recovery period.

## CRediT authorship contribution statement

Zachary Blashinsky: Writing the paper, data analysis and interpretation, data collection, study design.

Edit the paper.

Steven latta: Writing the paper, data analysis and interpretation, data collection, study design. Edit the

paper.

Luis Vargas: Study design, treating physician, data collection, manuscript edit.

Schurhoff: Study design, image analysis, data collection, manuscript edit

Zvijac: Study design, treating physician, data collection, manuscript edit.

Uribe: Study design, treating physician, data collection, manuscript edit.

## Ethical approval

IRB approval was obtained and has been included in the submission packet.

Committee: The Baptist Health South Florida IRB

Address:8900 North Kendal Drive, Miami, FL, 33176

Protocol Number: 11-046

Date of Approval: January 29, 2024

## Registration of research studies

N/A,

## Consent

Patient consent was obtained from the patient to publish this work and utilize images and data collected. Written consent is available upon request.

## Funding

No funding was disclosed by the authors.

## Guarantor

Dr. Luis Vargas

## Declaration of competing interest

The authors, their immediate families, and any research foundation with which they are affiliated have not received any financial payments or other benefits from any commercial entity related to the subject of this article.

The study was conducted under Institutional review board approval for long-term follow-up of patients treated with inlay total shoulder arthroplasty.
